# Secondary Structural Preferences of Some Antibacterial Cyclooctapeptides in the Presence of Calcium(II)

**DOI:** 10.1155/2012/730239

**Published:** 2012-12-18

**Authors:** Tarshona Stevens, Nykia McNeil, Xiuli Lin, Maria Ngu-Schwemlein

**Affiliations:** Department of Chemistry, Winston-Salem State University, Winston-Salem, NC 27110, USA

## Abstract

The purpose of this study is to understand the interactions of some antibacterial cationic amphipathic cyclooctapeptides with calcium(II) and their secondary structural preferences. The thermodynamic parameters associated with calcium(II) interactions, between the antibacterial active cyclooctapeptides (**COP 1–6**) and those that did not exhibit significant activities (**COP 7–9**), were studied by isothermal titration calorimetry. Calcium(II) binding in the absence and presence of micellar dodecylphosphocholine (DPC), a membrane mimicking detergent, was conducted by circular dichroism (CD). Both groups of cyclopeptides showed weak binding affinities for calcium(II) (*K_b_ ca*. 10^−3^ M^−1^). However, CD data showed that the antimicrobial peptides **COP 1–6** adopted a twisted beta-sheet structure (positive CD absorption band at *ca*. 203 nm) in the presence of calcium(II) in micellar DPC. In contrast, **COP 7–9**, which lacked antibacterial activity, adopted a different conformational structure (negative CD absorption band at *ca*. 203 nm). These results indicate that these cyclopeptides could adopt secondary structural preferences in the presence of calcium(II) amidst a hydrophobic environment to elicit their antibacterial activity. These findings could be useful in facilitating the design of cyclopeptide derivatives that can adopt this beta-sheet-like secondary structure and, thereby, provide a useful molecular template for crafting antibacterial compounds.

## 1. Introduction

Antimicrobial peptides (AMP) are a promising type of antibacterials [1, 2]. These compounds usually share a common site of action: the bacterial membrane. They usually exhibit strong selectivity toward the target bacterial membranes and kill rapidly. Amongst this class of compounds are the polycationic alpha helical peptides such as gramicidin A [3], melittin [4], cecropin [5], and magainin [6, 7], which play a significant role in host defense against bacteria. Other attractive antimicrobial candidates from this class are the macrocyclic peptides, which also target bacterial membranes and could elude bacterial resistance.

Interest in cyclic peptides began following the discovery of gramicidin S, a cyclic decapeptide antibiotic produced by a strain of *Bacillus brevis* [8]. Hodges et al. synthesized analogues of gramicidin S and examined the role of hydrophobicity in microbial specificity. Although they found that the therapeutic window could be optimized for each type of bacteria by modulation of peptide hydrophobicity, their results showed that the structure and antimicrobial activity relationship is intricate and diverse [9, 10] and, hence, requires further studies. Other groups have also explored head-to-tail cyclization of peptides for biological activity because they have lower conformational entropies and more defined conformations when compared to their linear congeners. Some cyclic peptides also exhibit more selective target recognition and reduced biodegradability by some proteases [11]. These features make cyclopeptides attractive candidates for the design of new antimicrobial agents. Fernandez-Lopez et al. at the Scripps Research Institute reported that cyclic peptides with an even number of alternating D- and L-amino acids can adopt a stable, ring-shaped, flat structure, which can stack to form nanotubes due to the participation of peptide backbone-backbone intermolecular hydrogen bonding. They have shown that these cyclic peptides can self-assemble and embed in a lipid bilayer membrane to form an efficient transmembrane ion channel [12]. However, there is a need to better understand the mechanism of action, particularly at the bacterial membrane and its associated divalent metal ions (Ca^2+^ and Mg^2+^). These divalent ions play a critical role in maintaining the integrity of the bacterial membrane. A structure-calcium binding and antimicrobial activities study would be worthwhile to gain some insights of the significance of calcium(II) associations with antimicrobial cyclooctapeptides.

In this study we prepared some cationic amphipathic cyclooctapeptides by microwave-assisted solid-phase peptide synthesis and evaluated their *in vitro* antimicrobial activities. Subsequently we assessed their binding affinity for calcium ions and their associated secondary structural changes. The objective of this study is to gain some understanding of the interaction of cationic amphipathic cyclooctapeptides with hydrophobic bacterial membranes and their associated divalent metal ions such as calcium(II). Within this frame of study, we investigated the thermodynamic parameters associated with calcium(II) binding by using isothermal titration microcalorimetry (ITC) and evaluated the conformational changes of the cyclopeptides upon calcium(II) binding by circular dichroism (CD).

## 2. Experimental

### 2.1. Materials and Methods

All chemicals were obtained from commercial suppliers and used without further purification. Fmoc-Glu(Wang Resin LL)-*O*Dmab (4-{*N*-[1-(4,4-dimethyl-2,6-dioxocyclohexylidene)-3-methylbutyl]amino}benzyl ester) resin and Fmoc-L- and D-amino acids containing the following side chain protecting groups: Cys(Trt), His(Trt), Lys(Boc), and Glu(t-Bu) were purchased from EMD Chemicals, Inc. (Gibbstown, NJ). 2-[(1*H*-Benzotriazol-1-yl)-1,1,3,3-tetramethyluronium hexafluorophosphate (HBTU) and N-hydroxybenzotriazole (HOBT) were also purchased from EMD Chemicals, Inc. (San Diego, CA, USA). Diisopropylethylamine (DIEA), collidine, triisopropylsilane (TIS), 3,6-dioxa-1,8-octanedithiol (DODT), trifluoroacetic acid (TFA), diisopropylcarbodiimide (DIPCDI), (7-azabenzotriazol-1-yloxy)tripyrrolidinophosphonium hexafluorophosphate (PyAOP), 1-hydroxy-7-azabenzotriazole (HOAT), and dichloromethane (DCM) were obtained from Sigma-Aldrich (St. Louis, MO, USA). *N*,*N*-Dimethylformamide (DMF) and anhydrous ether were obtained from VWR (West Chester, PA, USA). HPLC grade water, acetonitrile, and isopropanol were purchased from Fisher Scientific (Pittsburgh, PA, USA). Reversed-phase HPLC columns (Vydac 218TP54 and 218TP510, 300 Å, 5 *μ*m) were purchased from Chrom Tech, Inc. (Apple Valley, MN, USA). **COP 1** and **COP 2** were prepared as described previously [13]. **COP 3** was obtained from AnaSpec, Inc. (Fremont, CA, USA).

### 2.2. Preparation of **COP 4–9**

**COP 4–9** were prepared by microwave-assisted solid-phase peptide synthesis following the standard Fmoc-strategy by using the CEM Discovery microwave peptide synthesizer. Fmoc-Glu(Wang Resin LL)-*O*Dmab resin (0.33 meq/g substitution, 0.289 g, 0.1 mmol) was loaded onto a 25 mL polypropylene reaction tube fitted with a fiber-optic temperature probe for controlling the microwave power, polyethylene filter, and a Teflon seal ball. Deprotections were performed with 20% piperidine and 0.1 M HOBT in DMF solution for 4 minutes at 26 W with a maximum temperature of 75°C. All couplings were performed in the presence of a 5-fold molar excess of Fmoc-protected amino acids dissolved in HBTU : HOBT : DIEA : Fmoc-protected amino acid (0.9 : 1 : 2 : 1) in DMF. Coupling reactions were conducted under microwave irradiation at 21 W for 6 minutes with a maximum temperature of 75°C. Coupling conditions of cysteine and histidine residues were conducted with DIPCDI/HOBT (1 : 1) (5 eq. with respect to peptide-resin) in DMF (preactivation time 5 min) for 8 min at 16 W with a maximum temperature of 50°C following published procedures [14, 15]. After *N*-terminal Fmoc deprotection, the Dmab protecting group was removed with 5% hydrazine in DMF under microwave irradiation at 30 W for 10 minutes with a maximum temperature of 75°C [16, 17]. This step was repeated twice with freshly prepared 5% hydrazine in DMF. The peptide resin was then washed successively with DMF (3 × 5 mL) and dichloromethane (3 × 5 mL). On-resin cyclization was performed by mixing the peptidyl resin with a solution of PyAOP (0.5 mmol), HOAT (0.5 mmol), and DIEA (2.0 mmol) in 4 mL DMF (3 × 20 min at 75°C). The cyclopeptidyl resin was then washed successively with DMF (3 × 5 mL), dichloromethane (3 × 5 mL), methanol (1 mL), and dried overnight under high vacuum. Final cleavage of the cyclopeptide from the resin, and deprotections of the amino acid side chain protecting groups were carried out with a solution of TFA/H_2_O/TIS/DODT (9.25 : 0.25 : 0.25 : 0.25) (6 mL) at 11 W for 40 min with a maximum temperature of 38°C. Following cleavage, the cyclopeptide was precipitated with ice-cold anhydrous ether (40 mL). The suspension was kept at −10°C for 16 hrs and then centrifuged. The cyclopeptide pellet was washed with cold ether (3 × 40 mL) and the pellet was dried under high vacuum overnight. 

The crude peptides were analyzed by HPLC on a C-18 reversed-phase Vydac column (218TP54, 300 Å, 5 *μ*m, 4 mm × 250 mm) with a Rainin Dynamax system configured with two Dynamax 300 pumps and a Dynamax dual-wavelength detector interfaced with the Rainin Star LC Chromatography Workstation. The peptides were purified on a semipreparative C-18 reversed-phase Vydac column (Vydac 218TP54, 300 Å, 5 *μ*m, 10 mm × 250 mm). The mobile phase was H_2_O/0.1% trifluoroacetic acid (TFA) (A), and CH_3_CN (50%)/isopropanol (50%)/0.08% TFA (B) delivered by the Rainin Dynamax 300 HPLC system with UV monitoring at 214 nm. The analytical HPLC condition was 10% B to 60% B over 30 min at a flow rate of 1 mL/min, whereas the semipreparative HPLC condition was 10% to 40% over 30 min with a flow rate of 5 mL. The retention time for each cyclopeptide is as shown in Table 1. All peptides were at least 95% pure by HPLC. The overall percent yield for the purified peptides for the eighteen-step solid-phase peptide synthesis varied from 15% to 20%. The purified peptides were characterized by electrospray ionization mass spectrometry at the Mass Spectrometry Facility, Georgia State University. The observed mass for the molecular ion, [M]^+^, corresponds to the calculated value as shown in Table 1.

### 2.3. Antimicrobial Susceptibility Testing (Mueller-Hinton Broth)

Microbiological studies were performed on *Escherichia coli* ATCC 25922 and *Staphylococcus aureus* ATCC 10566 (American Type Culture Collection (ATCC), Rockville, MD, USA) using carefully standardized conditions [18]. A suspension of the bacteria (in exponential phase of growth) was prepared and then diluted to yield a final concentration of approximately 1 × 10^5^ colony forming units (cfu) per milliliter. Stock peptides were dissolved in 9% sucrose solutions containing 5% dimethyl sulfoxide. The 96-well microtiter tray containing the bacterial cultures and peptides was incubated for 18 hrs at 35°C. From these serial dilution tests, the minimum inhibitory concentration (MIC) of the peptides, which is the lowest test concentration of peptides that completely inhibits growth of the bacteria, was determined by visual examination of the wells for turbidity. All tests were conducted in triplicate and the reported MIC values were derived from at least three independent experiments. When experimental MIC values for each peptide vary, they do not vary by more than one test peptide concentration, and the test was repeated.

### 2.4. Isothermal Titration Calorimetry

Microcalorimetric titrations of peptides with metal ions were conducted by isothermal titration microcalorimetry (ITC) using a Microcal VP-ITC Instrument (Northampton, MA, USA). Experiments were carried out at 30°C in 10% acetonitrile in water. The peptide concentration was 0.15 or 0.30 mM (1.34 mL sample cell), and the metal ion [Ca(ClO_4_)_2_] concentration was varied from 9 to 15 mM in the syringe. Automated titrations were conducted until saturation, up to a Ca^2+^/peptide mole ratio of about 12 : 1. Heats of dilution and mixing for each experiment were measured by titrating calcium(II) solution into 10% acetonitrile solution. The effective heat of each peptide and calcium(II) interaction was corrected for dilution and mixing effects by subtracting the enthalpy change derived from the titration of calcium(II) solution into the 10% acetonitrile solution without the peptide. In a similar way, ITC experiments were carried out at 30°C in 10% aqueous acetonitrile containing 10 mM DPC. These heats of bimolecular interactions were obtained by integrating the peak following each injection of calcium(II). The binding isotherms were derived from the raw ITC data following correction for dilution and mixing effects. These isotherms were fitted using the one-site model by a nonlinear least square analysis [19, 20] with Microcal Origin 7.0 software (Microcal Software, Inc., Northampton, MA, USA) to determine the molar enthalpy change for binding, Δ*H*, and the corresponding binding constant, *K*_*b*_. From these values, the thermodynamic characterization of the interaction at 30°C was determined from the fundamental equations of thermodynamics, Δ*G* = −*RT* ln *K*_*b*_ and Δ*S* = (Δ*H* − Δ*G*)/*T*.

### 2.5. Structural Studies by Circular Dichroism

CD measurements were carried out on a Jasco J-815 Circular Dichroism Spectrometer (Easton, MD, USA) equipped with a Peltier temperature-controlled cell holder (PTC-423S/C). Spectra were recorded using 0.328 mM peptide solutions by sampling every 1 nm with an averaging time of 1 s, employing a 0.1 cm path length quartz cell. Each spectrum represents an average of four consecutive scans measured at 25°C, which was corrected by subtracting a corresponding blank solution. Stock dodecylphosphocholine (DPC) was prepared as 0.6 M solutions in 10% acetonitrile in water. Concentrate stock solution of calcium perchlorate (1.0 or 5.0 M) in 10% acetonitrile was titrated into the peptide solution (0.328 mM) to maintain various mole ratios of calcium ion to peptide, up to a molar ratio of 60. The CD spectrum was recorded following a one-minute equilibration after each titration at incremental molar ratios of 5 or 10. The changes in molar ellipticity (deg M^−1^ cm^−1^ per residue) at specific wavelengths were determined using the spectral analysis platform in the Jasco Spectra Manager I program (Jasco software, Inc.).

## 3. Results and Discussion

### 3.1. Design and Synthesis of Cyclooctapeptides

Some cationic amphipathic cyclooctapeptides were prepared by microwave-assisted solid-phase peptide synthesis, and their *in vitro* antimicrobial activities were evaluated for this study. Cyclooctapeptides with alternating L- and D-amino acid residues have been previously reported by Fernandez-Lopez et al. to exhibit antibacterial activities [21]. Therefore, we designed and prepared some similar but cationic amphipathic cyclooctapeptides with the intention to achieve a range of antibacterial activities for this study. These cyclopeptides **COP 1–3** (Table 2) were designed to introduce a single amino acid residue substitution at the junction between the hydrophobic and hydrophilic residues of the primary peptide structure, consisting of a leucine (**COP 1**), tryptophan (**COP 2**), or cysteine (**COP 3**). **COP 4–9** were designed to include a glutamyl residue to reduce the overall cationic charges on these amphipathic cyclopeptides. They were prepared by utilizing microwave energy to enhance each step in the solid-phase synthesis of the linear octapeptide as well as the on-resin head-to-tail cyclization. We have chosen *O*-Dmab (4{4*N*-[1-(4,4-dimethyl-2,6-dioxocyclohexylidene)-3-methylbutyl]-amino} benzyl ester) [16] over the *O*-allyl esters [22] as an orthogonal *α*-carboxyl protecting group of Glu because of its ease in removal by hydrazine under microwave irradiation. In general, on-resin synthesis of these cyclooctapeptides was achieved by a three-step process as previously reported by CEM, Inc. [17], with minor changes as described in the experimental section. It is well documented that cysteine, histidine, and aspartic acid are susceptible to racemization during microwave solid-phase peptide synthesis. Accordingly, precautions were taken to minimize racemization during the synthesis of those cyclopeptides containing cysteine and histidine by lowering the microwave coupling temperature from 75°C to 50°C as described by Palasek et al. [14] by using the coupling method reported by Angell et al. [15]. Crude peptides were purified to at least 95% purity by reversed-phase high-performance liquid chromatography and electrospray ionization mass spectrometry analysis was used to confirm the expected molecular mass (Table 1). 

### 3.2. Antimicrobial Activity

The minimum inhibitory concentration (MIC) of peptides was generally determined in the range of concentrations from 256 *μ*g/mL to 2 *μ*g/mL using the standard Muller-Hinton microbroth dilution antimicrobial susceptibility tests for aerobic bacteria, as recommended by the Clinical and Laboratory Standards Institute [18]. The test peptides **COP 1** and **2** showed similar antimicrobial activity toward both Gram-negative (*E. coli*) and Gram-positive (*S. aureus*) bacteria, with MIC of 16 *μ*g/mL and 8 *μ*g/mL, respectively (Table 2). However, changing one of the hydrophobic amino acids (e.g., Leu or Trp) to a more hydrophilic residue, Cys (**COP 3**), did not lead to any significant change in the MIC values when compared to **COP 1** and **2**. **COP 4–9** were then designed to include a glutamyl residue to reduce the overall cationic charges on these amphipathic cyclopeptides. Although **COP 4** did not exhibit any change in activity against *E. coli* (Table 2), its activity against *S. aureus* decreased by two fold. A lysine residue in **COP 4** was sequentially substituted with histidine in **COP 5**, cysteine in **COP 6**, and leucine in **COP 7**. Although **COP 5** exhibited a fourfold decrease in activity against *E. coli*, its antimicrobial activity for *E. coli* was reduced by half when compared to **COP 4**. As the net positive charges of the cyclopeptide decreased, antimicrobial activity decreased by fourfold (see **COP 6**). Further decrease in net positive charges resulted in the loss of antimicrobial activities (**COP 7–9**). The decreasing antimicrobial activities of these cyclopeptides could be attributed to their diminishing cationic character and an inadequate balance of amphipathic residues in their sequences. These structurally similar cyclopeptides (**COP 1–9**) were subsequently evaluated for calcium(II) binding affinity by ITC and assessed for conformational changes upon calcium(II) binding by circular dichroism (CD).

### 3.3. Isothermal Calorimetric Studies

**Figure 1:**
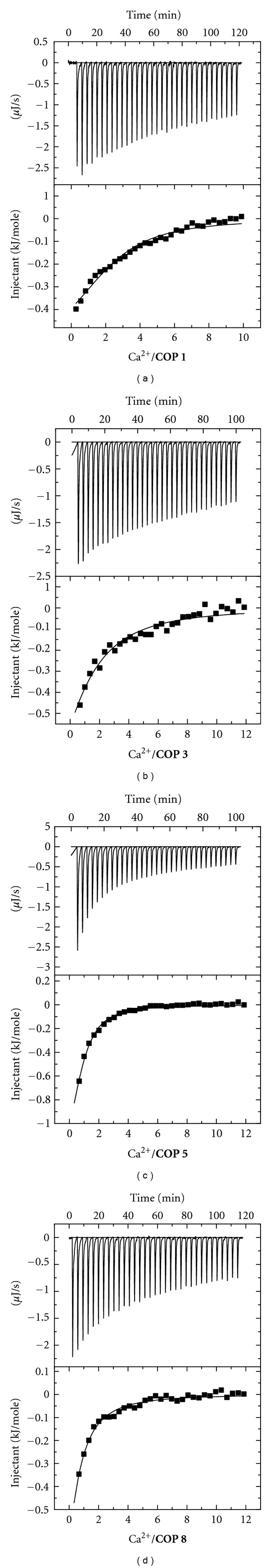
ITC data of (a) **COP 1**, (b) **COP 3**, (c) **COP 5**, and (d) **COP 8** following titration with Ca^2+^. Raw ITC titration data (top panels). Binding isotherms (bottom panels) are derived from the data in the corresponding top panels following correction for dilution and mixing effects.

Some representative ITC data for the interactions of cyclooctapeptides with calcium(II) are shown in Figure 1. The ITC titration data (top panels) show the total measured heat associated with each titration of calcium(II), normalized by the molar ratio of Ca^2+^/**COP** in the calorimeter cell. These interactions are moderately exothermic and therefore the heats for the association isotherms (bottom panels) are consequently low. From these binding isotherms, the corresponding thermodynamic parameters for the interactions between the cyclopeptides and calcium(II) were determined to derive the corresponding binding constants, *K*_*b*_. The calculated thermodynamic parameters and binding affinity values of these cyclopeptides (**COP 1–9**) for calcium(II) are as shown in Table 3. They showed weak binding affinities for calcium(II) (*K*_*b*_ values range from *ca*. 3.1 × 10^3^ to 8.0 × 10^3^ M^−1^). Their binding affinities for calcium(II) are similar despite their differences in antibacterial activities. Their binding enthalpy change values are negative (Δ*H* values range from *ca*. −0.5 to −11.3 kJ mol^−1^ for **COP 1–9**), whereas the associated entropy change values are positive (Δ*S* values range from *ca*. 28.9 to 67.8 JK^−1^ mol^−1^). Essentially, all these associations are both enthalpically and entropically favored. As shown in Table 1, there is entropy/enthalpy compensation in some cases. Calcium binding by **CP 4** and **CP 6**, which do not show much antibacterial activities, is relatively a more exothermic process compared to calcium binding by other cyclopeptides. Correspondingly, calcium binding by **CP 1–3**, which exhibit higher antibacterial activities, is more entropically driven. However, this entropy compensation is also demonstrated by the inactive peptides (**CP 7–9**).

In order to mimic the hydrophobic environment of the bacterial membrane that antimicrobial peptides commonly target, the interactions of these cyclopeptides with calcium(II) were also conducted in the presence of micellar dodecylphosphocholine (DPC). The calculated thermodynamic parameters for these interactions are shown in Table 3. Although their binding affinity for calcium(II) (*K*_*b*_ values range from *ca*. 3.3 × 10^3^ to 5.4 × 10^3^ M^−1^) is similar to that observed in the absence of micellar DPC, their interaction with calcium(II) is slightly less exothermic (Δ*H* values range from *ca*. −0.8 to −5.0 kJ mol^−1^ for **COP 3–9**). However, their association is compensated by an increase in entropy (Δ*S* values range from *ca*. 42.7 to 66.5 JK^−1^ mol^−1^). This entropy/enthalpy compensation could be attributed to a greater degree of dehydration of the hydration spheres around the cyclopeptide molecules and calcium ions following complex formation in the hydrophobic micellar DPC environment. However, there is no significant trend in the enthalpy or entropy changes following calcium(II) binding in the presence of micellar DPC. Likewise, there is no correlation between these thermodynamic parameters associated with calcium(II) binding by cyclopeptides that show antibacterial activities versus those that do not show much activity.

The above results show that the binding affinities of cyclooctapeptides (**COP 1–9**) for calcium(II) are similar, despite slight differences in the thermodynamic parameters associated with their underlying bimolecular interactions. These binding affinities are also not significantly affected by the presence of micellar DPC, which show that amphipathic cyclopeptides could coassemble with bacterial membrane and simultaneously bind calcium(II). These results show that varying the combination of cationic and hydrophobic residues in these cyclooctapeptides does not change their binding affinities for calcium(II). Additionally, incorporating one or more glutamyl residues does not enhance calcium(II) binding by these cyclopeptides. Based on these observations, we postulate that the carboxyl oxygen atoms of the cyclopeptide backbone may be the main donor atoms for calcium(II) association.

### 3.4. Circular Dichroism Studies

Peptides that adopt a specific peptide backbone-folding pattern (secondary structure) will show a characteristic CD spectrum [23, 24]. Accordingly, CD spectroscopy is a convenient method for monitoring conformational changes in the peptide backbone resulting from interactions with other molecules or metal ions. 

**Figure 2:**
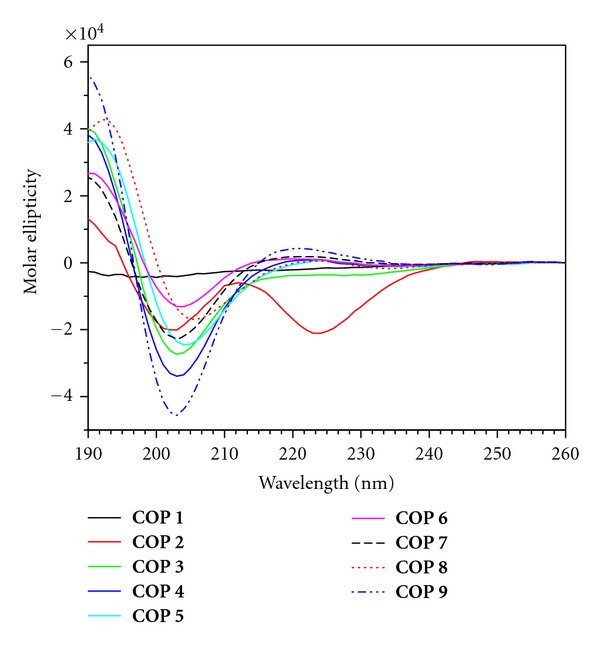
CD spectra of **COP 1–9.**

The CD of **COP 1** and **COP 2** were previously reported by us [13] in another study. **COP 1** adopted an unordered structure whereas **COP 2** adopted a weakly twisted partial beta-sheet structure, typified by having n*π*∗ and *π* *π*∗ bands of similar amplitudes (two minima at 201 nm and 222 nm with similar magnitude). CD data show that **COP 3–9** generally exhibit a very weak negative absorption band at *ca*. 235 nm, a weak positive band near 222 nm (n*π*∗ transition), a strong negative band near 200 nm, and a strong positive band just below 190 nm (first *π* *π*∗ transition), which is reminiscent of an unordered conformation (Figure 2) [25]. The unstructured peptide backbone structure could be attributed to the high composition of ionized lysine and glutamate residues in these cyclooctapeptides.

**Figure 3:**
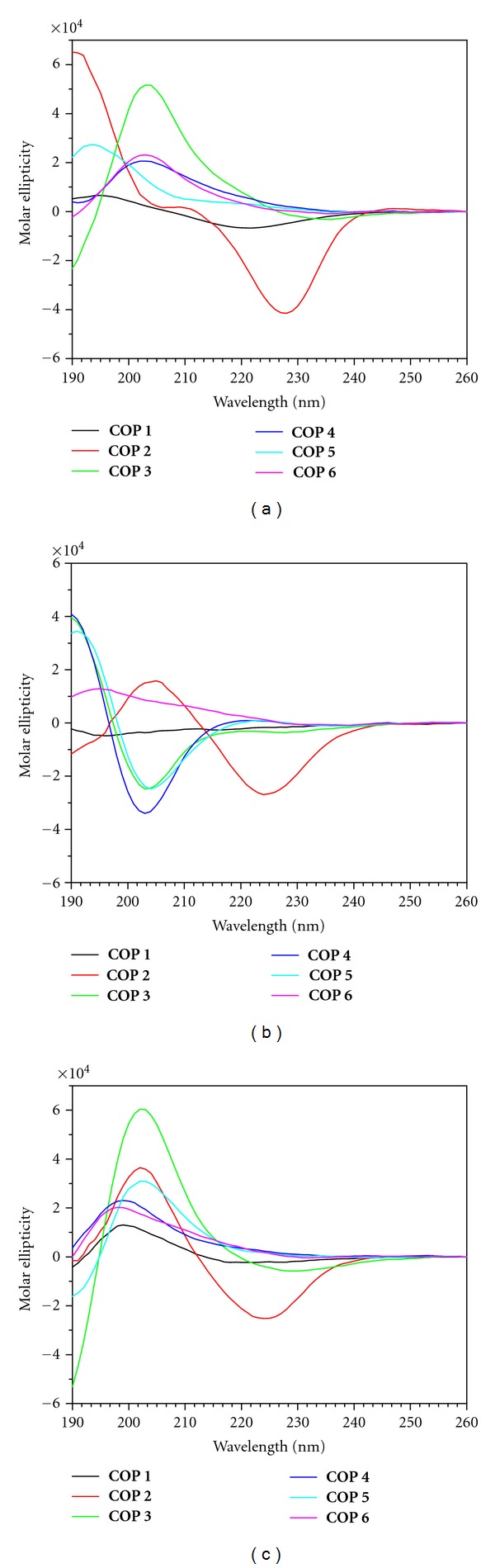
CD spectra of **COP 1–6** in (a) 100 mM Ca^2+^ only, (b) in 10 mM DPC only, and (c) in 10 mM DPC following titrations with Ca^2+^ at a Ca^2+^/**COP** ratio of 50.

**Figure 4:**
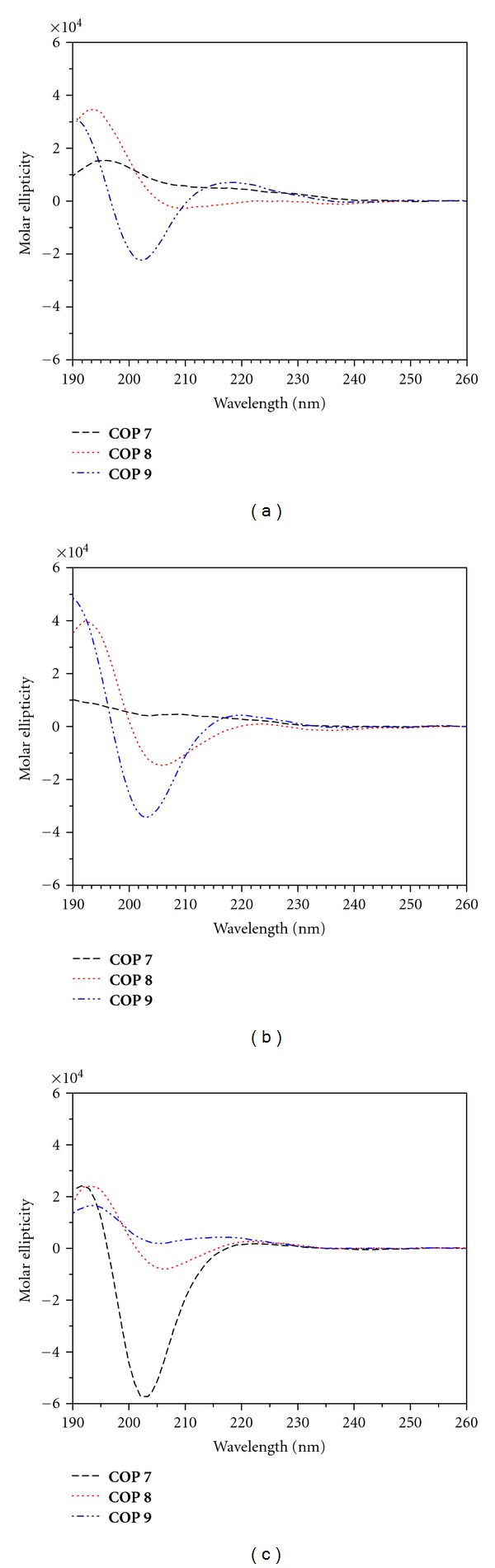
CD spectra of **COP 7–9** in (a) 100 mM Ca^2+^ only, (b) in 10 mM DPC only, and (c) in 10 mM DPC following titrations with Ca^2+^ at a Ca^2+^/**COP** ratio of 60.

In the presence of 100 mM calcium(II) (Figure 3(a)), the CD spectra of the antibacterial active cyclopeptides (**COP 1–6**) indicated that they undergo conformational changes. The negative band at *ca*. 202 nm transitioned into positive bands with maximum intensity at various wavelengths. The CD of the inactive cyclopeptides (**COP 7-8**) also showed similar changes although **COP 9** did not exhibit significant changes in its CD profile (Figure 4(a)).

In order to mimic the hydrophobic environment of bacterial membrane, the CD of the cyclopeptides were conducted in the presence of micellar dodecylphosphocholine (10 mM DPC). In the presence of micellar DPC (Figure 3(b)), the antibacterial peptides **COP 1**and **3–5** did not show any significant CD changes. However, **COP 2** undergoes a structural transition in the presence of micellar DPC as previously reported [13]. It shows a conformational change from that of a partial *β*-sheet to one with predominantly *β*-sheet structure, typified by n*π*∗ and *π* *π*∗ transitions of opposite signs but with approximately equal magnitude. **COP 6** also showed a change in its CD profile. Its weak negative band at 230 nm increased in intensity to form a positive band shifted to 195 nm. On the other hand, the CD of the inactive cyclopeptides (**COP 8-9**) did not change in the presence of micellar DPC (Figure 4(b)).

Remarkably, in the presence of both micellar DPC and 16.5 mM calcium(II) (Ca^2+^/peptide molar ratio of 50), all of the antimicrobial peptides (**COP 1–6**) share a similar CD profile: they adopted a strongly twisted beta sheet- or turn-like structure (strong positive band at *ca*. 203 nm, *π* *π*∗ transition) (Figure 3(c)). In contrast, **COP 7–9**, which lacked antimicrobial activity, did not exhibit the same CD profile. Instead, the CD of these cyclopeptides in the presence of both micellar DPC and calcium(II) showed a negative band, ranging from 203 to 206 nm (Figure 4(c)). This common CD profile exhibited by **COP 7–9** were not observed in the presence of calcium(II) (Figure 4(a)) or micellar DPC (Figure 4(b)).

The above conformational analysis by CD shows that a single amino acid substitution in these amphipathic cyclooctapeptides did not change their secondary structure of the peptide backbone significantly (Figure 2). Correspondingly, varying the combination of cationic and hydrophobic residues in these cyclopeptides did not affect their conformation. However, the antimicrobial peptides, **COP 1–6**, share a similar CD profile in the presence of both calcium(II) and micellar DPC (Figure 3(c)), that is, remarkably different from those that lack antimicrobial activity (**COP 7–9**) (Figure 4(c)).

## 4. Conclusion

The purpose of this work is to better understand the interactions and secondary structural preferences of some antibacterial cationic amphipathic cyclooctapeptides with calcium(II). The ITC results of this study show that the binding affinity of these cyclopeptides for calcium(II) do not select for antibacterial activities. However, the secondary structure of these cyclopeptides in the hydrophobic bacterial milieu enriched with calcium(II) could have a significant role in killing bacteria. In this study, the conformational preferences of **COP 1–9** were evaluated by circular dichroism (CD). The CD data showed that the antibacterial active cyclopeptides (**COP 1–6**) adopted a twisted beta-sheet structure (positive CD absorption band at *ca*. 203 nm) in the presence of calcium(II) in micellar DPC. In contrast, cyclopeptides **COP 7–9**, which lacked antibacterial activity, adopted a different secondary structure (negative CD absorption band at *ca*. 203 nm). Although these cyclopeptides share many similar amino acid residues, their propensity to exhibit antibacterial activity are distinguished by their secondary structure in the presence of both calcium(II) and micellar detergent (DPC). While calcium(II) is necessary for eliciting the cyclopeptide conformation for their antibacterial activity, its binding affinity for these cyclopeptides does not select for antibacterial activity. The results from this study could be useful in facilitating the design of cyclopeptide derivatives that can adopt this beta-sheet-like secondary structure, and thereby provide a useful molecular template for crafting antibacterial compounds.

## Figures and Tables

**Table 1:** HPLC and mass spectrometry data of the synthesized cyclopeptides (**COP4–9**).

Test peptides		Retention time (min)	ESI-MS (*m*/*z*) [M]^+^
**COP 4**	c[d-Leu-Leu-d-Leu-Lys-d-Lys-Lys-d-Lys-Glu]	15.2	981.68 (981.27)^a^
**COP 5**	c[d-Leu-Leu-d-Leu-His-d-Lys-Lys-d-Lys-Glu]	15.4	990.68 (990.24)
**COP 6**	c[d-Leu-Leu-d-Leu-Cys-d-Lys-Lys-d-Lys-Glu]	18.2	956.61 (956.24)
**COP 7**	c[d-Leu-Leu-d-Leu-Leu-d-Lys-Lys-d-Lys-Glu]	20.2	966.88 (966.26)
**COP 8**	c[d-Leu-Leu-d-Leu-His-d-Lys-His-d-Lys-Glu]	15.1	999.80 (999.21)
**COP 9**	c[d-Leu-Leu-d-Leu-Glu-d-Lys-Lys-d-Lys-Glu]	16.8	982.80 (982.23)

^a^Calculated [M]^+^ values are shown in parenthesis.

**Table 2:** Antimicrobial susceptibility test results for **COP 1–9**.

Test peptides		*E*. *coli* ATCC 25922 MIC (*μ*g mL^−1^)	*S*. *aureus* ATCC 10566 MIC (*μ*g mL^−1^)
**COP 1**	c[Leu-d-Leu-Leu-d-**Leu**-Lys-d-Lys-Lys-d-Lys]	16	8
**COP 2**	c[Leu-d-Leu-Leu-d-**Trp**-Lys-d-Lys-Lys-d-Lys]	16	8
**COP 3**	c[d-Leu-Leu-d-Leu-**Cys**-d-Lys-Lys-d-Lys-Lys]	16	16
**COP 4**	c[d-Leu-Leu-d-Leu-**Lys**-d-Lys-Lys-d-Lys-Glu]	16	32
**COP 5**	c[d-Leu-Leu-d-Leu-**His**-d-Lys-Lys-d-Lys-Glu]	32	128
**COP 6**	c[d-Leu-Leu-d-Leu-**Cys**-d-Lys-Lys-d-Lys-Glu]	128	128
**COP 7**	c[d-Leu-Leu-d-Leu-**Leu**-d-Lys-Lys-d-Lys-Glu]	>256	>256
**COP 8**	c[d-Leu-Leu-d-Leu-**HIs**-d-Lys-**His**-d-Lys-Glu]	>256	>256
**COP 9**	c[d-Leu-Leu-d-Leu-**Glu**-d-Lys-Lys-d-Lys-Glu]	>256	>256

**Table 3:** Thermodynamic parameters data for Ca^2+^ binding to **COP 1–9**.

Test peptide	Binding affinity^a^ for Ca^2+^ *K*_*b*_ (M^−1^)	Change in enthalpy Δ*H* (kJ mol^−1^)	Change in Gibbs free energy Δ*G* (kJ mol^−1^)	Change in entropy Δ*S* (J K^−1^ mol^−1^)
**COP 1** ^ b^	(3.4 ± 0.6) × 10^3^	−0.5 ± 0.1	−20.5 ± 0.4	65.7 ± 1.7
**COP 2** ^ b^	(4.8 ± 1.3) × 10^3^	−0.6 ± 0.04	−20.9 ± 0.8	67.8 ± 3.8
**COP 3**	(7.9 ± 2.8) × 10^3^	−2.9 ± 1.7	−22.2 ± 1.3	63.6 ± 5.4
**COP 3** in DPC^c^	(3.3 ± 0.3) × 10^3^	−0.8 ± 0.04	−20.5 ± 0.2	64.0 ± 0.8
**COP 4**	(3.1 ± 0.7) × 10^3^	−11.3 ± 1.3	−20.1 ± 0.8	28.9 ± 4.6
**COP 4** in DPC	(4.7 ± 1.1) × 10^3^	−1.7 ± 0.1	−21.3 ± 0.8	64.9 ± 2.1
**COP 5**	(5.3 ± 1.4) × 10^3^	−2.5 ± 1.3	−21.3 ± 0.8	62.3 ± 6.3
**COP 5** in DPC	(5.4 ± 0.6) × 10^3^	−2.1 ± 0.1	−21.3 ± 0.4	63.6 ± 2.1
**COP 6**	(6.4 ± 2.8) × 10^3^	−10.0 ± 6.7	−21.8 ± 0.8	38.5 ± 23.8
**COP 6** in DPC	(4.9 ± 0.7) × 10^3^	−5.0 ± 2.1	−21.3 ± 0.4	54.4 ± 7.9
**COP 7**	(8.0 ± 1.2) × 10^3^	−3.3 ± 0.8	−22.6 ± 0.4	63.6 ± 3.8
**COP 7** in DPC	(4.7 ± 1.0) × 10^3^	−0.8 ± 0.4	−13.8 ± 7.5	42.7 ± 7.1
**COP 8**	(4.9 ± 0.3) × 10^3^	−2.9 ± 0.8	−21.3 ± 0.4	60.7 ± 0.8
**COP 8** in DPC	(4.4 ± 0.02) × 10^3^	−0.8 ± 0.1	−21.3 ± 0.04	66.5 ± 0.4
**COP 9**	(5.4 ± 1.2) × 10^3^	−3.3 ± 0.4	−21.3 ± 0.8	59.4 ± 2.1
**COP 9** in DPC	(4.1 ± 0.03) × 10^3^	−1.7 ± 0.2	−20.9 ± 0.04	63.6 ± 0.4

^a^Values correspond to the mean of three experiments and the standard error mean.

^b^Low heat changes for this association in DPC prevented meaningful calculation of the corresponding thermodynamic parameters with certainty.

^c^Micellar DPC solutions were prepared at 10 mM DPC.

## Abbreviations

t-Boc: Tertiary butyloxycarbonyl
DIEA: *N*,*N*′-Diisopropylethylamine
DIPCDI: *N*,*N*′-Diisopropylcarbodiimide
DODT: 3,6-Dioxa-1,8-octanedithiol
Fmoc: 9-Fluorenylmethyloxycarbonyl
Fmoc: AA *N*-9-Fluorenylmethyloxycarbony protected amino acid
HBTU: 2-[(1*H*-benzotriazol-1-yl)-1,1,3,3-tetramethyluronium hexafluorophosphate
HOAT: 1-Hydroxy-7-azabenzotriazole
HOBT: *N*-Hydroxybenzotriazole
Dmab: (4-{*N*-[1-(4,4-Dimethyl-2,6-dioxocyclohexylidene)-3-methylbutyl]amino}benzyl ester)
PyAOP: (7-Azabenzotriazol-1-yloxy)tripyrrolidinophosphonium hexafluorophosphate
t-Bu: Tertiary butyl
TFA: Trifluoroacetic acid
TIS: Triisopropylsilane
Trt: Trityl.
